# Smokers’ utilization of quitting methods and vaping during pregnancy: an empirical cluster analysis of 2016–2018 Pregnancy Risk Assessment Monitoring System (PRAMS) data in seven US states

**DOI:** 10.1186/s12884-023-05608-3

**Published:** 2023-05-02

**Authors:** Xi Wang, Nora L. Lee, Igor Burstyn

**Affiliations:** 1 PolicyLab, Children’s Hospital of Philadelphia, 2716 South Street, Philadelphia, PA 19146 USA; 2 Department of Epidemiology and Biostatistics, Drexel University, 3215 Market Street, Philadelphia, PA 19104 USA; 3 Department of Environmental and Occupational Health, Drexel University, 3215 Market Street, Philadelphia, PA 19104 USA

**Keywords:** Smoking, Cessation, Latent class analyses, Smoking status,, Quitting method, Electronic cigarettes, Pregnancy, Patterns

## Abstract

**Background:**

Patterns of utilization of numerous smoking cessation methods among pregnant women amidst the increasing popularity of vaping (use of e-cigarettes) remains unknown.

**Methods:**

This study included 3,154 mothers who self-reported smoking around the time of conception and delivered live births in 2016–2018 in seven US states. Latent class analysis was used to identify subgroups of smoking women based on their utilization of 10 surveyed quitting methods and vaping during pregnancy.

**Results:**

We identified four subgroups of smoking mothers with different utilization patterns of quitting methods during pregnancy: 22.0% reported “not trying to quit”; 61.4% tried to “quit on my own” without any behavioral or pharmacological assistance; 3.7% belonged to the “vaping” subgroup; and 12.9% utilized “wide-ranging methods” with higher use rate of multiple approaches, such as quit line and nicotine patch. Compared to mothers “not trying to quit,” the subgroup trying to “quit on my own” were more likely to be abstinent (adjusted OR 4.95, 95% CI 2.82–8.35) or to reduce the number of cigarettes smoked daily (adjusted OR 2.46, 95% CI 1.31–4.60) in late pregnancy, and these improvements lasted into early postpartum. We did not observe a measurable reduction in smoking among the “vaping” subgroup or women trying to quit with “wide-ranging methods”.

**Conclusions:**

We identified four subgroups of smoking mothers with different utilization patterns of eleven quitting methods during pregnancy. Pre-pregnancy smokers who tried to “quit on my own” were most likely to be abstinent or to reduce smoking amount.

**Supplementary Information:**

The online version contains supplementary material available at 10.1186/s12884-023-05608-3.

## Background

Smoking during pregnancy is a concern in the US; about 1 in 12 women who gave birth in 2018 reporting smoking during the 3 months before pregnancy, with most continuing to smoke during pregnancy [1]. Maternal smoking during pregnancy is consistently reported as a predictor of adverse birth outcomes, such as preterm birth and low birth weight, as well as fetal and infant mortality [2, 3].

Quitting smoking at any point during pregnancy is accepted as beneficial. Not surprisingly, more women quit smoking when they become pregnant than at any other time in their lives [4]. A range of interventions are available to help pregnant women stop smoking [4, 5]. The 2020 US Preventive Services Task Force (USPSTF) guidelines recommended behavioral interventions for smoking cessation in pregnant women. Based on pooled data from trials including over 26,000 women, behavioral interventions were more effective than usual care or minimal support for smoking cessation in late pregnancy (pooled risk ratio 1.35, 95% confidence interval (CI) 1.23–1.48) [6]. The 2020 USPSTF report concluded that there is limited evidence on the benefits and harms of pharmacotherapies in pregnancy, including nicotine replacement therapy (NRT), bupropion, and varenicline [6]. In spite of the existence of behavioral interventions and pharmacotherapies, studies among the general population show that the largest proportion of smokers successfully quit without any assistance (often referred to as the “cold turkey” approach) [7–14]. However, it remains unclear how often these cessation methods and the “cold turkey” approach are used by pregnant smoking people (i.e., utilization pattern) and how helpful these approaches are to achieving smoking abstinence in this special population (i.e., effectiveness).

Vaping (use of electronic cigarettes or e-cigarettes) provides nicotine mist by inhalation without intake of smoke. The 2018 National Academies of Sciences, Engineering, and Medicine (National Academies) report concluded that there was substantial evidence that except for nicotine, under typical conditions of use, exposure to potentially toxic substances from e-cigarettes is significantly lower compared with combustible tobacco cigarettes [15]. However, the 2020 USPSTF report concluded that the current evidence is insufficient to assess the balance of benefits and harms of e-cigarettes for tobacco cessation in pregnancy [6].

The utilization patterns of various smoking cessation methods among pregnant women in the face of increasing popularity of vaping in the last decade remains unknown. We aim to describe women’s patterns of utilizing smoking cessation methods and vaping during pregnancy, to investigate personal characteristics associated with these patterns, and to identify how these patterns relate to change in smoking amount in late pregnancy and early postpartum.

## Methods

### Data source and sample

We used data from the 2016–2018 Pregnancy Risk Assessment Monitoring System (PRAMS), a public health surveillance project conducted by the US Centers for Disease Control and Prevention (CDC) and state health departments to collect state-specific, population-based data. Details of its design and methodology are described elsewhere [16]. Briefly, PRAMS samples women who have had a recent live birth from the state’s birth certificates and uses a mixed mail and telephone survey to ask women about their behaviors and experiences before, during, and shortly after the index pregnancy. We restricted our sample to participants who self-reported smoking traditional (combustible) cigarettes in the 3 months before pregnancy and lived in the states of Arkansas, Georgia, Iowa, Utah, Virginia, Vermont, or West Virginia (because these seven states asked questions on use of quitting methods and vaping). Consequently, 3,154 mothers were included (weighted sample of 131,644 mothers). All PRAMS data were de-identified, and the analysis was deemed by the Institutional Review Board of Drexel University as being exempt from review.

### Measures

PRAMS participants were asked about the average quantities of cigarettes smoked daily (less than 1, 1–5, 6–10, 11–20, or > 20 cigarettes per day) during the 3 months before pregnancy (i.e., pre-pregnancy), during the last 3 months of pregnancy (i.e., late pregnancy), and at the time of survey completion (i.e., early postpartum) [17, 18]. By comparing each woman’s smoking behavior in pre-pregnancy to that in late pregnancy, we categorized pre-pregnancy smokers into three groups: 1) abstinent (completely stopped smoking), 2) reduced cigarettes amount smoked per day, and 3) not reduced cigarettes amount smoked per day. Similarly, we created three groups of pre-pregnancy smokers by making the same comparison to smoking behavior in early postpartum.

Women who self-identified as pre-pregnancy smokers were asked whether they used any of the following methods to help them quit during pregnancy (selecting all that apply): 1) Set a specific date to stop smoking; 2) Use booklets, videos, or other materials to help me quit; 3) Call a national or state quit line or go to a website; 4) Attend a class or program to stop smoking; 5) Go to counseling for help with quitting; 6) Use a nicotine patch, gum, lozenge, nasal spray or inhaler; 7) Take a pill like Zyban® (also known as Wellbutrin or Bupropion) to stop smoking; 8) Take a pill like Chantix® (also known as Varenicline) to stop smoking; 9) Try to quit on my own (e.g., cold turkey); or, 10) Other. Each co-author independently reviewed the open-ended responses for “other” and re-coded, whenever possible, to one of the other well-defined groups; any disagreement was resolved by the consensus of the authors. In addition, women were asked whether they used e-cigarettes during the last 3 months of pregnancy. E-cigarettes are defined as battery-powered devices that use nicotine liquid rather than tobacco leaves and produce vapor instead of smoke. While not included in the survey as a quit method, in this study we considered use of e-cigarettes as another approach to quitting, resulting in 11 non-mutually exclusive categories of quitting approaches a pre-pregnancy smoker could take during pregnancy in attempting to quit.

We extracted data on mother's demographic characteristics (age, educational level, race/ethnicity, marital status), parity (number of previous live births), previous preterm delivery, adequacy of prenatal care, maternal pre-pregnancy body mass index (BMI), and alcohol consumption before pregnancy (yes/no). We calculated an index of adequacy of prenatal care based on the initiation time and number of prenatal care visits, and categorized women as receiving intensive, adequate, intermediate, or inadequate prenatal care [19].

### Statistical analysis

Pre-pregnancy smokers were first divided into two general groups – the “not trying to quit” group who did not report using any of the 11 categories of quitting methods during pregnancy, and the group who used at least one of the quitting methods during pregnancy. Among the latter group, we employed latent class analysis (LCA) to empirically identify subgroups (latent classes) of these women based on their nuanced utilization patterns of the 11 quitting methods. LCA is a statistical method which posits that homogenous unobserved segments (latent classes) can be identified within a heterogeneous group using a set of observed variables [20]. To first determine the most appropriate number of latent classes in our sample, we fit models considering 2- to 5-segment solutions and selected the optimal solution based on the criteria of fit statistics, model identification, parsimony, and interpretability. Fit statistics included the Akaike Information Criterion (AIC), Bayesian Information Criterion (BIC), and sample size-adjusted BIC [21]. To assess model identification, we estimated the parameters for each model using 1,000 random starting values and measured the proportion of iterations that converged to the same maximum likelihood solution [20]. In addition, we reviewed the solutions in light of pre-existing knowledge and ease of their interpretation. After determining the optimal solution, we then assigned each respondent to the latent class for which they had the highest posterior probability of membership. Subsequent analyses were based on these quitting subgroups. We also developed a name for each identified latent class that concisely captured its most salient and distinct features. SAS PROC LCA add-on was used for LCA [21].

We described the socio-demographic (age, educational level, race/ethnicity, marital status), obstetric (parity, previous preterm delivery, adequacy of prenatal care, pre-pregnancy BMI), and behavioral (alcohol consumption before pregnancy) features of the overall sample and of each quitting subgroup though cross-tabulations. Multivariable logistic models were also used to assess the associations of membership in quitting subgroup with change in smoking amount in late pregnancy and early postpartum periods, after adjusting for the above-mentioned socio-demographic, obstetric, and behavioral confounders, in terms of adjusted odds ratios (aOR) and 95% CI. In all these analyses, we applied statistical weighting schemes to account for different sampling rates in different strata and nonresponse in PRAMS data [16].

## Results

### Characteristics of smoking mothers in 2016–2018 PRAMS

Table 1 shows the characteristics of a state-representative population-based sample (from seven US states) of 3,154 women who smoked prior to pregnancy. The smoking mothers vary in the average cigarettes smoked daily in the 3 months before pregnancy, with 29% reporting smoking 1–5 cigarettes/day, 29% smoking 6–10 cigarettes/day, and 25% smoking 11–20 cigarettes/day. Most of the smoking mothers in the sample were White (71%), had an education level of high school or below (58%), and were not married (62%). The majority (76%) of the smoking mothers received at least adequate prenatal care during their sampled pregnancy.

**Table 1:** Characteristics of smoking mothers in 2016–2018 PRAMS in seven US states

	**n**	**Weighted column %**
**Total**	3154	
**# of cigarettes smoked per day during the 3 months before pregnancy**		
Less than 1 cig/day	247	10.3
1–5 cigs/day	789	28.9
6–10 cigs/day	895	28.8
11–20 cigs/day	899	25.4
> 20 cigs/day	324	6.6
**Mother’s age (years)**		
< 20	216	4.9
20–24	870	28.9
25–29	1052	35.4
30–34	661	22.3
35 +	354	8.5
**Mother’s education**		
9–12 Grade, no diploma	588	15.5
High school grad/GED	1335	42.7
Some college, no degree/associate degree	939	31.6
Bachelor’s/master’s/doctorate/prof	255	10.2
**Mother’s race/ethnicity**		
Non-Hispanic White	1826	71.4
Non-Hispanic Black	460	15.6
Hispanic	170	5.2
Others/Unknown	698	7.8
**Marital status**		
Married	1062	38.2
Other	2080	61.8
**Number of prior live births**		
0	1194	38.7
1	899	28.8
> = 2	1052	32.5
**History of preterm birth**		
Yes	207	3.2
No	2934	96.8
**Pre-pregnancy body mass index (BMI)**		
Underweight (< 18.5 kg/m^2^)	198	4.3
Normal (18.5–24.9 kg/m^2^)	1229	38.6
Overweight (25.0–29.9 kg/m^2^)	696	27.3
Obese (30.0 + kg/m^2^)	952	29.8
**Kotelchuck index for prenatal care**		
Inadequate	489	14.5
Intermediate	268	9.6
Adequate or intensive	2329	75.9
**Drink alcohol before pregnancy**		
No	1226	33.3
Yes	1878	66.7
**Birth year**		
2016	1260	33.2
2017	1047	36.9
2018	847	29.9

### Overall utilization rates of quitting approaches among smoking mothers

Table 2 presents the use rate of the 11 quitting approaches in the overall sample. The PRAMS survey option is to “check all that apply” (with e-cigarette use as a separate question), so categories are not mutually exclusive. While the majority of smoking mothers (78%) used at least one approach, most reported trying to quit on their own during pregnancy (71.5%). The second most popular quitting method was to set a specific date to stop smoking (25.2%). Few resorted to either NRT (6.3%) or e-cigarettes (6.9%). The use of pharmacological methods was uncommon. No common theme emerged from the open-ended responses from people who reported “Other” quitting approach (4.2% of the sample). For example, two persons answered “exercise”, two – “eat candy”, and one – “sleep a lot”. More than one-fifth (22.0%) of pre-pregnancy smokers did not use any of the 11 quitting approaches during pregnancy.

**Table 2:** Utilization of quitting methods in the smoking mothers from seven US states, overall and by subgroups

	**All** **pre-pregnancy smokers**	**Subgroups of smokers by utilization of quitting methods**			
		**“Not trying to quit”**	**Latent classes of women who tried to quit**		
			**“Quit on my own”** **(latent class 1)**	**“Vaping”** **(latent class 2)**	**“Wide-ranging methods” (latent class 3)**
	*(N* = *3154)*	*(N* = *677)*	*(N* = *1856)*	*(N* = *109)*	*(N* = *512)*
		*(weighted 22.0% of all pre-pregnancy smokers)*	*(weighted 61.4% of all pre-pregnancy smokers)*	*(weighted 3.7% of all pre-pregnancy smokers)*	*(weighted 12.9% of all pre-pregnancy smokers)*
**Quitting methods**	**Use rate of each quitting method (weighted %)**				
Set a specific date to stop smoking	25.2	0.0	23.4	0.0	83.8*
Use booklets, videos, or other materials to help me quit	6.2	0.0	0.0	0.0	47.8*
Call a national or state quit line or go to a website	2.7	0.0	1.2	0.5	15.0*
Attend a class or program to stop smoking	0.6	0.0	0.2	0.0	3.6*
Go to counseling for help with quitting	0.9	0.0	0.1	0.0	6.2*
Use a nicotine patch, gum, lozenge, nasal spray or inhaler	6.3	0.0	2.3	10.7	34.9*
Take a pill like Zyban® (also known as Wellbutrin® or Bupropion®) to stop smoking	1.3	0.0	0.5	12.6*	3.8
Take a pill like Chantix® (also known as Varenicline) to stop smoking	0.8	0.0	0.0	1.5	5.8*
Try to quit on my own (e.g., cold turkey)	71.5	0.0	100.0*	13.5	74.4
Electronic cigarettes	6.9	0.0	3.5	80.1*	13.4
Other	4.2	0.0	4.7	17.4*	5.3

^*^For each quitting method, the subgroup with the highest use rate was highlighted with *

### Latent classes of smoking mothers based on utilization of quitting approaches

LCA was applied to identify subgroups (latent classes) based on utilization patterns of the 2,477 women who used at least one of the quitting methods. The identification and fit statistics of 2- to 5-segment LCA solutions are shown in Supplementary Table S1. The 3-segment solution performed much better than the 2-segment solution on the measures of fit statistics (AIC and BIC) and identification, while the 4-segment solution only further improved the identification and fit statistics by a small degree. Based on the additional criteria of parsimony and interpretability, we chose the 3-segment solution as the optimal model in the subsequent analyses. Table 2 presents the use of the 11 quitting methods in each of the three identified latent classes. The “quit on my own” subgroup (*latent class 1*) constituted the largest subgroup of smoking women (61.4%) and was characterized by women who tried to quit on their own. No or very few women in this subgroup used booklets, attended class, or took medications like Zyban or Chantix to stop smoking. The “vaping” subgroup (*latent class 2*) constituted a small proportion (3.7%) of smoking mothers and represented those who had the highest use rate (80.1%) of e-cigarettes during pregnancy. This subgroup also had the highest use rate of medications. The subgroup that used “wide-ranging methods” (*latent class 3*) were mainly smoking mothers who had higher use rates of most quitting methods than other subgroups, and this subgroup constituted 12.9% of all smoking mothers. Eighty-four percent of smoking mothers in this subgroup set a specific date to stop smoking, 47.8% used booklets, videos or other materials, and 34.9% used nicotine patch, gum, lozenge, nasal spray or inhaler. This subgroup also had the second highest use rate of medications. In the following analyses, in addition to these three subgroups (“quit on my own”, “vaping”, and “wide-ranging methods”) identified from women who tried at least one quitting methods, the remaining 677 (22.0%) pre-pregnancy smokers who did not use any of the 11 quitting methods were included as a separate “not trying to quit” subgroup.

### Characteristics associated with smoking mothers’ utilization of quitting methods

In Supplementary Table S2, we present smoking women’s demographic, obstetric, and behavioral characteristics by their membership in each latent class based on their utilization of the 11 quitting approaches. We observed that the intensity of smoking before pregnancy was positively associated with women utilizing multiple quitting methods. Among the four subgroups of smoking mothers, the “not trying to quit” group had the highest proportion (14.6%) of smoking less than 1 cigarette daily before pregnancy, followed by the group trying to “quit on my own” (10.3%). The group utilizing “wide-ranging methods” of quitting had the highest proportions of smoking 11 or more (44.1%). The “vaping” subgroup was younger, with 25.9% of them under 20 years old and 32.4% age 24–24 years.

### Utilization of quitting approaches during pregnancy and change in smoking amount in late pregnancy and early postpartum

Table 3 presents the changes in average daily smoking amount between pre-pregnancy and late pregnancy among smoking mothers grouped by their utilization of quitting methods. Overall, 1,414 (55.3%) of pre-pregnancy smokers self-reported to be abstinent in late pregnancy. We found that smoking mothers who tried to “quit on my own” were more likely to completely quit smoking (become abstinent) or reduce the number of cigarettes smoked per day in late pregnancy, compared to those who did not report trying to quit. Forty-four percent of the smoking mothers “not trying to quit” were abstinent in late pregnancy, while 64.8% of the smoking mothers who tried to “quit on my own” became abstinent. In adjusted models, the subgroup trying to “quit on my own” were on average 4.95 (95% CI 2.82–8.35) times more likely to be abstinent and 2.46 (95% CI 1.31–4.60) times more likely to reduce smoking amount in late pregnancy, compared to the “not trying to quit” subgroup. The “vaping” subgroup had lower rates of being abstinent (31.3% vs 44.3%) or reducing smoking amount (30.2% vs 34.2%) in late pregnancy than those who did not try to quit, but the differences between these two subgroups were not apparent in the adjusted models. The subgroup of smoking mothers who tried to quit with “wide-ranging methods” had a lower rate of being abstinent than the “not trying to quit” subgroup (35.7% vs 44.3%), but a higher rate of reducing smoking amount (46.8% vs 34.2%); however, the effect estimates for this subgroup were imprecise in the adjusted regression models.

**Table 3:** Adjusted associations between utilization of quitting approaches and changes in smoking among pre-pregnancy smokers in late pregnancy, in 2016–2018 PRAMS participants in seven states

Subgroups of pre-pregnancy smokers by use of quitting methods	N	Smoking amount in *late pregnancy*					
		**Weighted row %**			**Adjusted odds ratio** (95% confidence interval) *		
		**Abstinent**	**Reduced amount compared to pre-pregnancy**	**Not reduced**	**(Odds of abstinent) / (Odds of not reduced)**	**(Odds of reduced) / (Odds of not reduced)**	**(Odds of abstinent or reduced) / (Odds of not reduced)**
“Not trying to quit”	677	44.3	34.2	21.4	reference	reference	reference
“Quit on my own”	1856	64.8	24.8	10.5	4.85 (2.82–8.35)	2.46 (1.31–4.60)	3.02 (1.85–4.92)
“Vaping”	109	31.3	30.2	38.5	0.62 (0.23–1.70)	0.43 (0.14–1.35)	0.54 (0.23–1.31)
“Wide-ranging methods”	512	35.7	46.8	17.5	1.76 (0.73–4.25)	1.80 (0.84–3.89)	1.53 (0.77–3.04)

^*^Adjusted for average cigarette numbers smoked per day in the 3 months before pregnancy, mother’s age, education level, race/ethnicity, marital status, previous preterm history, plurality (number of previous live births), Kotelchuck index of prenatal care, pre-pregnancy BMI, drinking alcohol before pregnancy, and birth year. Statistical weighting schemes were applied in regression models to account for different sampling rates in different strata and nonresponse in PRAMS data

In Table 4, we examined the changes in average daily smoking amount from pre-pregnancy to early postpartum among smoking mothers grouped by their utilization of quitting methods. Overall, 955 (39.1% of the whole sample) pre-pregnancy smokers self-reported to be abstinent in early postpartum. The lower abstinence rate in early postpartum compared to late pregnancy was primarily due to 575 (18.8% of the whole sample, 37.3% of the group) people who quit in late pregnancy and became smokers againg in early postpartum. The abstinence rate of the “quit on my own” group was 44.6% in early postpartum, still higher than the other three subgroups. In the adjusted model, compared to the “not trying to quit” subgroup, the “quit on my own” subgroup was on average 1.89 (95% CI 1.18–3.03) times more likely to be abstinent in early postpartum.

**Table 4:** Adjusted associations between utilization of quitting approaches and changes in smoking among pre-pregnancy smokers in early postpartum, in 2016–2018 PRAMS participants in seven states

Subgroups of pre-pregnancy smokers by use of quitting methods	N	Smoking amount in *early postpartum*					
		**Weighted row %**			**Adjusted odds ratio** (95% confidence interval) *		
		**Abstinent**	**Reduced amount compared to pre-pregnancy**	**Not reduced**	**(Odds of abstinent) / (Odds of not reduced)**	**(Odds of reduced) / (Odds of not reduced)**	**(Odds of abstinent or reduced) / (Odds of not reduced)**
“Not trying to quit”	677	36.5	20.9	42.7	reference	reference	reference
“Quit on my own”	1856	44.6	21.2	34.2	1.89 (1.18–3.03)	1.58 (0.91–2.75)	1.74 (1.16–2.60)
“Vaping”	109	31.2	21.1	47.7	1.25 (0.42–3.73)	0.67 (0.24–1.86)	0.89 (0.39–2.02)
“Wide-ranging methods”	512	18.9	26.2	54.9	0.49 (0.22–1.07)	0.86 (0.42–1.78)	0.68 (0.38–1.22)

^*^Adjusted for average cigarette numbers smoked per day in the 3 months before pregnancy, mother’s age, education level, race/ethnicity, marital status, previous preterm history, plurality (number of previous live births), Kotelchuck index of prenatal care, pre-pregnancy BMI, drinking alcohol before pregnancy, and birth year. Statistical weighting schemes were applied in regression models to account for different sampling rates in different strata and nonresponse in PRAMS data

## Discussion

### Uptake of various quitting methods during pregnancy

In a large state-representative population-based sample of self-identified smoking women from seven US states, we found that smoking mothers are heterogeneous in their utilization of quitting methods during pregnancy. More than a fifth (22.0%) of smoking mothers did not report that they tried to quit during pregnancy and did not utilize any of 11 quitting approaches. The smoking mothers who tried to quit exhibited one of the three different patterns based on their utilization of quitting approaches: the majority (61.4%) tried to “quit on my own” without utilizing any counselling, assistive materials, or medications;12.9% utilized “wide-ranging methods” in trying to quit and reported relatively high use rate of most quitting methods, including using booklets or videos, setting a specific date, calling a quit line, and using nicotine replacement therapies; and the minority (3.7%) belonged to the “vaping” subgroup with higher use of e-cigarettes than the others.

### Smoking outcomes associated with utilization of quitting methods during pregnancy

We compared the changes in smoking amount during pregnancy by women’s utilization of quitting methods. We found that pre-pregnancy smokers who tried to quit using “wide-ranging methods” did not have significantly higher odds of abstinence or reduction in smoking amount compared to the smoking pregnant women who did not try to quit. Although it is hard to reconcile this with the reported efficacy of quitting methods from randomized clinical trials (RCTs), our finding is similar to the pattern reported by other observational studies [8, 22]. For example, in analyzing about 30,000 US smokers, Shiffman et al. found that smokers who sought any behavioral or pharmacologic treatments were less likely to be abstinent (OR = 0.75; 95% CI = 0.67–0.84) than those tried to quit without treatment, and those who sought multiple treatments were even less likely to be abstinent (e.g., smokers who used four or more treatments were less likely to be abstinent than those without any treatment, with OR of 0.60, 95% CI 0.43–0.85) [8].

We interpret our finding with several considerations. First, the efficacy of certain quitting methods is not as well established in pregnant populations as in the general population. For example, in 2020 the USFSTF conducted a meta-analysis of five placebo-controlled trials on the effectiveness of NRT for pregnant smoking women and generated a pooled effect of NRT on validated smoking cessation (pooled RR 1.11, 95% CI 0.79–1.56) [6]. Second, even if some RCTs reported efficacy, the real-world effectiveness of certain quitting approaches may be different. For example, most smokers who use NRT do so for short periods of time or at lower-than-recommended doses and therefore may not achieve the optimal outcomes observed in RCTs. Third, the fact that more-dependent smokers or the heaviest smokers were more likely to utilize multiple quitting methods may cause confounding by indication [23]. Even though we adjusted for pre-pregnancy smoking amount in the regression models, there might be residual confounding of this factor that may explain the seemingly counter-intuitive worse outcomes of the group who used wide-ranging quitting approaches.

We did not observe any measurable reduction in smoking among women who were vaping. A 2021 Cochrane review summarized 61 completed studies in the general population and found moderate-certainty evidence that e-cigarettes with nicotine increase quit rates compared to NRT, while the evidence comparing e-cigarettes with usual care/no treatment is less certain [24]. Admittedly, the subgroup of smoking women who use vaping as the main quitting approach during pregnancy was a minority of our sample and vaping among smoking women remains rare. Thus, the question of benefits of vaping during pregnancy through reduction of smoking is poorly illuminated by our analysis and will require either larger or more focused studies to evaluate.

In our sample, pre-pregnancy smokers who tried to “quit on my own” were most likely to be abstinent or to reduce average daily cigarettes smoked in late pregnancy, and these improvements lasted into early postpartum. Previous studies in the general population have consistently shown that a large majority of smokers who permanently stop smoking do so without any form of assistance [7–14, 25]. In 2010, Chapman et al. reviewed hundreds of studies on cessation interventions, and they concluded that up to three-quarters of ex-smokers have quit without assistance (“cold turkey” or cut down then quit) and unaided cessations is the most common method used by most successful ex-smokers [22]. In a US national longitudinal study using data from the Tobacco Use Supplement to the Current Population Survey (TUS-CPS) published in 2012, lighter smokers who tried to quit unassisted were 37% more likely to be successful than were those who used help, and heavy smokers who tried to quit unassisted had a 50% higher success rate than those who used help [26]. Although current clinical guidelines recommend that smokers be advised to use behavioral interventions or pharmacotherapies in quitting [6], researchers have suggested that overmedicalizing smoking cessation may disempower smokers and create artificial barriers to quitting [22]. Our study of a large sample of women who had live births across seven US states confirms the higher success rate of unassisted quitting during pregnancy. This finding highlights the opportunity for health care workers and public health authorities to present this empowering message to pregnant smokers who intend to try to quit.

### Strengths

This study used population-based data that were representative of all women who recently had live births in 2016–2018 from seven states in the US. LCA was applied to create latent classes of smoking women based on their utilization of 11 quitting methods during pregnancy. The LCA identified three latent classes and therefore avoided using arbitrary definitions of quitting patterns, which would be based on the many possible combinations of the 11 quitting approaches (in theory, there would be 2^11^ = 2,048 possible combinations).

### Limitations

In this study, smoking women’s use of quitting methods during pregnancy and their change in cigarettes smoked per day were self-reported in a postpartum survey. Smoking women may tend to over-report their use of quitting methods due to social desirability, which would introduce misclassification into our study. It is also important to note that due to concerns about confounding by indication and errors in reported behaviors, our findings are not to be interpreted as asserting causal relationships. In addition, our findings on the use rate of e-cigarettes using 2016–2018 PRAMS data may not reflect changes in more recent years. Evidence suggests that the US prevalence of daily e-cigarette use increased from 2017 to 2020 by about 1%, mainly among young adults aged 18 to 24 years and pregnant women [27]. The perception of harm of vaping likely evolved over time, as did the technology of e-cigarettes. Lastly, although we restricted our sample to participants who self-reported smoking in the 3 months before pregnancy and adjusted for average number of cigarettes smoked per day in that period, we did not have information on smoking history prior to the 3 months before pregnancy. Evidence suggests a narrow range for the initiation age of smoking—nearly 9 out of 10 adults who smoke cigarettes daily started smoking by age 18, and 99% started smoking by age 26 [28]. Therefore, the unmeasured confounding caused by duration of smoking history may have been partially accounted for in our analysis as we controlled for age.

## Conclusions

In a large representative population-based sample of smoking mothers from seven US states, we identified four subgroups of smoking mothers based on their different patterns of utilization of 11 quitting methods during pregnancy: the “not trying to quit” subgroup who reported not using any of the quitting approaches; a subgroup who tried to “quit on my own” without the assistance of other behavioral or pharmacological approaches; the “vaping” subgroup who had a high use rate of e-cigarettes; and a subgroup who utilized “wide-ranging methods” in attempting to quit. In our sample, pre-pregnancy smokers who tried to “quit on my own” were most likely to be abstinent or to reduce smoking amount in late pregnancy, and these improvements lasted into early postpartum. Our findings highlight the opportunity for presenting the positive message about unassisted cessation as the most common and successful quitting method to pregnant smokers who are ready to try to quit.

## Supplementary Information

**Additional file 1: Supplementary Table S1.** Latent Class Analysis: Fit Statistics by Number of Classes, among 2477 smoking mothers who tried >=1 quitting approach. **Supplementary Table S2.** Socio-demographic, obstetric and behavioral characteristics of the subgroups of smoking mothers clustered based on their utilization of quitting methods, in smoking mothers in 2016-2018 PRAMS in seven US states.

## Data Availability

The PRAMS data used in this study was provided from the PRAMS working groups and the Centers for Disease Control and Prevention (CDC). Per CDC PRAMS External Researcher Data Sharing Agreement, we are not allowed to release our analytic data set, and we are not allowed to use the data to conduct analyses other than those described in our proposal. Researchers may request PRAMS data for their studies by submitting a proposal to CDC (https://www.cdc.gov/prams/prams-data/researchers.htm).

## Abbreviations

USPSTF: United States Preventive Services Task Force
NRT: Nicotine replacement therapy
CDC: Centers for Disease Control and Prevention
PRAMS: Pregnancy Risk Assessment Monitoring System
LCA: Latent class analysis
AIC: Akaike Information Criterion
BIC: Bayesian Information Criterion
