# Supplementary material for: Smokers’ utilization of quitting methods and vaping during pregnancy: an empirical cluster analysis of 2016–2018 Pregnancy Risk Assessment Monitoring System (PRAMS) data in seven US states
Source: BMC Pregnancy Childbirth. 2023 May 2;23:306. doi: 10.1186/s12884-023-05608-3 (PMC10152601; doi:10.1186/s12884-023-05608-3)
Supplement: Supplementary file 1 — Additional file 1: Supplementary Table S1. Latent Class Analysis: Fit Statistics by Number of Classes, among 2477 smoking mothers who tried >=1 quitting approach. Supplementary Table S2. Socio-demographic, obstetric and behavioral characteristics of the subgroups of smoking mothers clustered based on their utilization of quitting methods, in smoking mothers in 2016-2018 PRAMS in seven US states. [file 12884_2023_5608_MOESM1_ESM.docx]

**Supplementary Table S1** Latent Class Analysis: Fit Statistics by Number of Classes, among 2477 smoking mothers who tried >=1 quitting approach

| **Number of classes** | **AIC*** | **BIC*** | **Adjusted BIC*** | **Degree of Freedom** | **Identification**** |
| --- | --- | --- | --- | --- | --- |
| 2 | 927.42 | 1061.16 | 988.08 | 2024 | 35.6% |
| 3 | 639.73 | 843.25 | 732.05 | 2012 | 79.7% |
| 4 | 532.33 | 805.63 | 656.30 | 2000 | 87.8% |
| 5 | 498.16 | 841.23 | 653.77 | 1988 | 1.4% |

* AIC Akaike Information Criterion, BIC Bayesian Information Criterion. (The lower, the better)

** The proportion of iterations (with 1000 starting values) that converged to the same maximum likelihood solution. (The higher, the better)

**Supplementary Table S2** Socio-demographic, obstetric and behavioral characteristics of the subgroups of smoking mothers clustered based on their utilization of quitting methods, in smoking mothers in 2016-2018 PRAMS in seven US states

|  | **Weighted column % in each subgroup** | | | | **P-value from Rao-Scott Chi-Square test** |
| --- | --- | --- | --- | --- | --- |
|  | **‘Not-trying-to-quit’ subgroup** | **“Quit on my own” subgroup** | **“Vaping” subgroup** | **“Wide-ranging methods” subgroup** |  |
|  | *(N=677)* | *(N=1856)* | *(N=109)* | *(N=512)* |  |
| **# of cigarettes smoked per day during the 3 months before pregnancy** |  |  |  |  |  |
| Less than 1 cigs/day | *14.6* | 10.3 | 0.8 | 5.4 | *0.0046* |
| 1-5 cigs/day | 26.7 | *31.3* | 23.4 | 22.7 |  |
| 6-10 cigs/day | 21.9 | 31.2 | *34.8* | 27.9 |  |
| 11-20 cigs/day | 30.0 | 21.0 | 34.5 | *35.8* |  |
| >20 cigs/day | 6.8 | 6.2 | 6.6 | *8.3* |  |
| **Mother’s age (years)** |  |  |  |  |  |
| <20 | 4.8 | 4.9 | *5.9* | 4.9 | 0.0490 |
| 20-24 | 20.7 | 32.0 | *32.4* | 27.2 |  |
| 25-29 | 33.9 | 35.6 | 25.4 | *39.5* |  |
| 30-34 | 29.1 | 19.8 | *34.4* | 19.1 |  |
| 35+ | *11.6* | 7.6 | 1.9 | 9.3 |  |
| **Mother’s education** |  |  |  |  |  |
| 9-12 Grade, No Diploma | 19.5 | 12.6 | *24.9* | 19.4 | *0.0088* |
| High School Grad/Ged | 32.7 | 45.1 | 40.7 | *48.9* |  |
| Some college, no degree/associate degree | *33.5* | 31.9 | 32.4 | 26.8 |  |
| Bachelors/masters/doctorate/prof | *14.4* | 10.4 | 2.1 | 4.9 |  |
| **Mother’s race/ethnicity** |  |  |  |  |  |
| Non-Hispanic White | 72.7 | 71.7 | 70.8 | 67.4 | 0.2675 |
| Non-Hispanic Black | 14.7 | 15.8 | 15.8 | 16.5 |  |
| Hispanic | 6.7 | 5.5 | 1.8 | 2.2 |  |
| Others/Unknown | 5.9 | 6.9 | 11.7 | 13.9 |  |
| **Marital status** |  |  |  |  |  |
| Married | 44.6 | 37.7 | 32.0 | 31.2 | 0.1468 |
| Other | 55.4 | 62.3 | 68.0 | 68.8 |  |
| **Number of Prior Live Births** |  |  |  |  |  |
| 0 | 32.1 | 41.7 | 42.6 | 34.5 | 0.4229 |
| 1 | 32.1 | 27.8 | 26.6 | 28.9 |  |
| >=2 | 35.7 | 30.5 | 30.8 | 36.6 |  |
| **History of Preterm Birth** |  |  |  |  |  |
| Yes | 2.4 | 2.9 | 5.2 | 5.1 | 0.2504 |
| No | 97.6 | 97.1 | 94.8 | 94.9 |  |
| **Pre-pregnancy body mass index (BMI)** |  |  |  |  |  |
| Underweight (< 18.5 kg/m^2^) | 2.6 | 4.2 | 8.7 | 6.6 | 0.1837 |
| Normal (18.5-24.9 kg/m^2^) | 41.0 | 37.1 | 29.7 | 44.3 |  |
| Overweight (25.0-29.9 kg/m^2^) | 28.8 | 26.0 | 39.8 | 27.2 |  |
| Obese (30.0+ kg/m^2^) | 27.7 | 32.7 | 21.7 | 21.9 |  |
| **Kotelchuck index for prenatal care** |  |  |  |  |  |
| Inadequate | 17.7 | 11.7 | *25.1* | 19.2 | *0.0034* |
| Intermediate | 8.3 | 8.0 | *20.1* | 16.1 |  |
| Adequate or intensive | 74.0 | *80.3* | 54.8 | 64.7 |  |
| **Drink alcohol before pregnancy** |  |  |  |  |  |
| No | 35.3 | 29.4 | *53.3* | 43.1 | *0.0049* |
| Yes | 64.7 | *70.6* | 46.7 | 56.9 |  |
| **Birth Year** |  |  |  |  |  |
| 2016 | 27.1 | 36.6 | 26.3 | 29.5 | 0.1697 |
| 2017 | 36.6 | 35.5 | 42.4 | 42.5 |  |
| 2018 | 36.3 | 27.9 | 31.3 | 28.0 |  |
